# Making the most of mortalities: Novel host-parasite records in a sandy inland mouse (*Pseudomys hermannsburgensis*)^☆^

**DOI:** 10.1016/j.ijppaw.2025.101037

**Published:** 2025-01-07

**Authors:** Fiona Knox, Nahiid Stephens, Sarah Keatley, Amanda Ash, James Douch, Saul Cowen, Kelly Rayner, Rebecca Vaughan-Higgins

**Affiliations:** a Murdoch University School of Veterinary Medicine, 90 South St, Murdoch, Western Australia, 6150, Australia; b Western Australia Department of Biodiversity Conservation and Attractions, Biodiversity and Conservation Science, Wildlife Place, Woodvale, Western Australia, 6026, Australia; c Melbourne University Department of Veterinary Biosciences, Asia-Pacific Centre for Animal Health, Parkville, Victoria, 3052, Australia; d School of Biological Sciences, The University of Western Australia, Crawley, Western Australia, 6009, Australia

**Keywords:** *Rodentolepis*, reintroduction, tapeworm, wildlife cancer, One Health, sarcoptic mange

## Abstract

From 2020 to 2022, systematic investigation of wildlife mortalities on Dirk Hartog Island, Western Australia was initiated to inform wildlife disease risk analyses for translocation purposes. As part of this monitoring, in November 2020, a sandy inland mouse (*Pseudomys hermannsburgensis*) was found deceased with multiple comorbidities. Gross necropsy, histopathology and ancillary molecular testing identified several novel host-parasite associations. *Sarcoptes scabiei* was identified via molecular methods in association with consistent cutaneous pathology, representing the first known detection of this parasite in an Australian native rodent. A putative novel virus belonging to the subfamily *Gammaherpesvirinae* was also identified, representing the first known detection of a herpesvirus (*Orthoherpesviridae*) from this species, although it was not clearly associated with other disease processes. A heavy burden of the cestode *Hymenolepis microstoma* was also present in the gastrointestinal tract, representing a new host record for this species, whilst a pancreatic adenocarcinoma was also found. Beyond the novelty of these host records, these findings contribute to important health baselines of rodent populations on Dirk Hartog Island and highlight the value of investigating mortalities and implementing health surveillance as part of ecological monitoring and wildlife translocation projects.

## Highlights

• *Sarcoptes scabiei* is reported for the first time in a wild Australian rodent.
• The detection of a putative novel herpesvirus is also described.
• The cestode *Hymenolepis microstoma* and a pancreatic adenocarcinoma were also found.
• The report shows the importance of health surveillance in wildlife translocations.

## Graphical abstract

**Figure:**
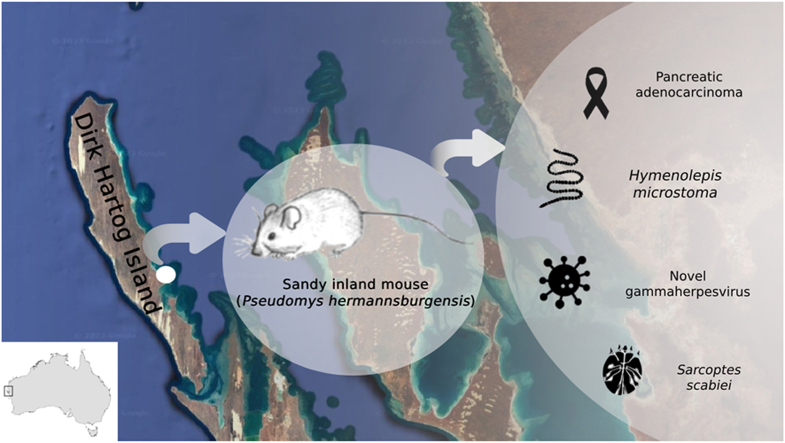

## 1 Introduction

Wildlife translocations are increasingly utilised in Australia to conserve threatened species, restore ecosystems or for wildlife salvage purposes (Morris et al., 2015). These management actions are associated with biosecurity risks, with the potential for exposure of naïve hosts to novel pathogens leading to potentially significant repercussions, ranging from individual to ecosystem-level effects (Kock et al., 2010; Wildlife Health Australia, 2018; Beckmann et al., 2022). To identify disease risk mitigation strategies, the Dirk Hartog Island National Park Ecological Restoration Project has routinely incorporated wildlife disease risk analyses (WDRAs) into translocation planning.

Historically utilised as a pastoral lease since the 1860s for sheep (*Ovis aries*) and goats (*Capra hircus*), Dirk Hartog Island (DHI) was established as a national park in 2009, setting the stage for ecological restoration (Algar et al., 2020), which aims to restore DHI to an ecological condition analogous to that observed when the first Europeans landed in 1616. By 2018, the Department of Biosecurity, Conservation and Attractions (DBCA) had achieved the goal of removing feral cats (*Felis catus*), sheep and goats from the island (Cowen et al., 2023). Subsequently, only four non-volant mammals remained: the ash-grey mouse (*Pseudomys albocinereus*), sandy inland mouse (*Pseudomys hermannsburgensis*), little long-tailed dunnart (*Sminthopsis dolichura*), and the introduced house mouse (*Mus musculus*; Algar et al., 2020; Cowen et al., 2023). This facilitated the commencement of wildlife translocations, which ultimately seek to establish 13 additional mammal or bird species on DHI, either as reintroductions or conservation translocations. This includes four native Australian rodent species: greater stick-nest rats (*Leporillus conditor*), Shark Bay mice (*Pseudomys gouldii*), heath mice (*Pseudomys shortridgei*), and desert mice (*Pseudomys desertor*; Cowen et al., 2023).

WDRAs frequently encounter knowledge deficiencies, including those related to disease hazards (both infectious and non-infectious) present in destination environments, posing challenges to informed decision making. To address this deficiency, a project commenced in 2020 to evaluate the baseline health of DHI's extant rodents before rodent translocations commenced. This project incorporated necropsy and routine histopathology on all rodent mortalities with additional targeted sampling for selected probable pathogens between 2020 and 2022. Sandy inland mice were one of the target species for this project. Sandy inland mice are a small (∼12g), social, omnivorous burrowing rodent species found throughout the semi-arid and arid regions of Australia (Dickman et al., 2010; Yip and Dickman, 2023). Although sandy inland mice are a common Australian rodent, to the authors' knowledge, there have been no known systematic health surveys of this species in Australia and few parasites have been described.

As part of the health surveillance project implemented on DHI, a sandy inland mouse suffering multiple co-morbidities was found deceased during ecological monitoring activities. The present study describes the multiple novel host-parasite associations detected during investigation of this mortality.

## 2 Materials and methods

**Fig. 1:**
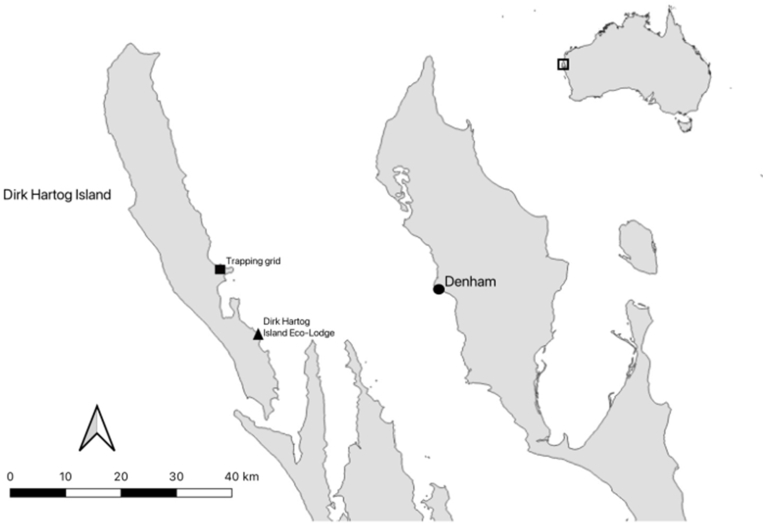
**The sandy inland mouse was found in a trapping grid set up for post-release monitoring of Shark Bay bandicoots on Dirk Hartog Island**. The trapping grid is in the Herald Bay region of Dirk Hartog Island. This area is approximately 15 km from the Dirk Hartog Island Eco-Lodge, where the only known domestic animals on the island are located. The nearest township is Denham, a small population on the mainland in the Shark Bay region of Western Australia.

In November 2020, a sandy inland mouse was found deceased in a Sheffield cage trap during post-translocation monitoring of Shark Bay bandicoots (*Perameles bougainville*) on DHI (Fig. 1). Traps were set in the late afternoon and baited with universal bait (peanut butter and oats) and cleared within 3 h of sunrise the following morning. Trapping was conducted as approved by the DBCA animal ethics committee (2019–23A) and tissue collection was approved by the Murdoch University ethics committee (Cadaver713).

A detailed physical examination, sample collection, and post-mortem examination of the deceased sandy inland mouse was conducted on-site by a veterinarian. Body condition was determined as described in Ullman-Culleré and Foltz (1999). Swabs were collected by swabbing of the oral, conjunctival, urogenital, and rectal regions using sterile flocked swabs (COPAN, Brescia, Italy). A combined urogenital-rectal and oral-conjunctival swab was collected for *Orthoherpesviridae* testing as part of a routine sampling project to investigate *Orthoherpesviridae* diversity of rodents on DHI and placed in viral transport medium (PathWest, Mount Claremont, Western Australia), refrigerated at 2–8 °C for four days before being placed in storage at – 80 °C until further testing. Gross findings were documented, and representative samples of tissue collected in 10% neutral buffered formalin, and frozen sections stored at – 20 °C for four days before being placed in long-term storage at – 80 °C. Samples of endoparasites were placed in 70% ethanol.

### 2.1 Histological analysis

Pieces of formalin-fixed tissue were trimmed and processed in ethanol and xylene before paraffin embedding. Five μm sections of the paraffin blocks were made and stained with haematoxylin and eosin. Additional staining for some sections included Gram, Periodic acid-Schiff (PAS) and Ziehl–Neelsen (ZN) stains where indicated. Examination of slides was performed by a Board Certified veterinary pathologist (NS).

### 2.2 Pan-herpesvirus PCR

A pan-herpesvirus polymerase chain reaction (PCR) was performed as per methods described in Douch et al. (2022). Briefly, DNA was extracted from both swabs using a QIAamp Viral RNA Mini Kit (QIAGEN) according to manufacturer instructions. This kit can extract both DNA and RNA. A nested pan-herpesvirus PCR targeting a conserved region of the DNA polymerase (DPOL) catalytic subunit UL30 was utilised (Table S1; VanDevanter et al., 1996). Electrophoresis was performed using 2% w/v agarose gel stained with SYBR Safe (Thermo Fisher Scientific). Bands of the expected size (∼200bp) were purified, with a sequencing reaction performed using a BigDye Terminator v3.1 sequencing kit (Applied Biosystems, Foster city, CA, USA). The amplicon was then submitted for Sanger sequencing (Australian Genome Research Facility, Melbourne).

### 2.3 Cestode identification

Following initial morphological examination of the cestode that had been stored in 70% ethanol, species identification was performed using molecular techniques. DNA was isolated from a cestode segment using a DNeasy blood and tissue kit (QIAGEN) according to manufacturer instructions following overnight Proteinase K incubation for digestion at 56 °C.

Extracted cestode DNA was amplified at two mitochondrial gene regions, the small subunit of ribosomal RNA (rrnS) as per Trachsel et al. (2007), and the cytochrome *c* oxidase subunit 1 (cox1) as per Bowles et al. (1992) followed by electrophoresis using a 1.5% w/v agarose gel (Table S1). Resultant bands of the expected size (∼227bp and ∼440bp, respectively) were then purified and sequenced in both directions using an ABI Prism Dye Terminator Cycle Sequencing Kit (Applied Biosystems, Foster City, CA, USA) as per manufacturer instructions.

### 2.4 Sarcoptes scabiei PCR

DNA was extracted from the frozen skin sample using a DNeasy blood and tissue kit (QIAGEN) according to manufacturer instructions following a 1–3 h Proteinase K digestion at 56 °C.

Extracted DNA was amplified at the cox1 gene region using methods as per Andriantsoanirina et al. (2015), followed by electrophoresis using a 1.5% w/v agarose gel. A resultant band of the expected size (∼400bp) was then purified and sequenced in both directions using an ABI Prism Dye Terminator Cycle Sequencing Kit as per manufacturer instructions.

### 2.5 Sequence analysis

All nucleotide sequences were compared with published sequences in GenBank (National Center for Biotechnology Information (NCBI)), using the nucleotide basic local alignment search tool (BLASTn), and assessed for similarity with described species.

The cestode cox1 and herpesvirus sequences were translated into amino acids in MEGA11 prior to analysis (Molecular Evolutionary Genetics Analysis; Tamura et al., 2021). Additional sequences for alignment were obtained using BLASTn or the protein basic local alignment search tool and manual searches in GenBank. Obtained sequences were aligned and trimmed using multiple sequence comparison by log-expectation in MEGA11 with default settings. Best models for maximum-likelihood phylogenetic analysis were determined through the model function in MEGA11, selecting the model with the lowest Bayesian information criterion and Akaike information criterion corrected values. The appropriate model was then used to create each phylogenetic tree, with confidence in relationships evaluated by a bootstrap analysis with 100 replicates.

## 3 Results

### 3.1 Signalment and gross necropsy findings

The adult male sandy inland mouse was found deceased in lateral recumbency in a cage trap. No overt traumatic cause of death was identifiable. No other vertebrates were present in the trap. The absence of temporary marking indicated the animal had not previously been captured in the same trap session.

**Fig. 2:**
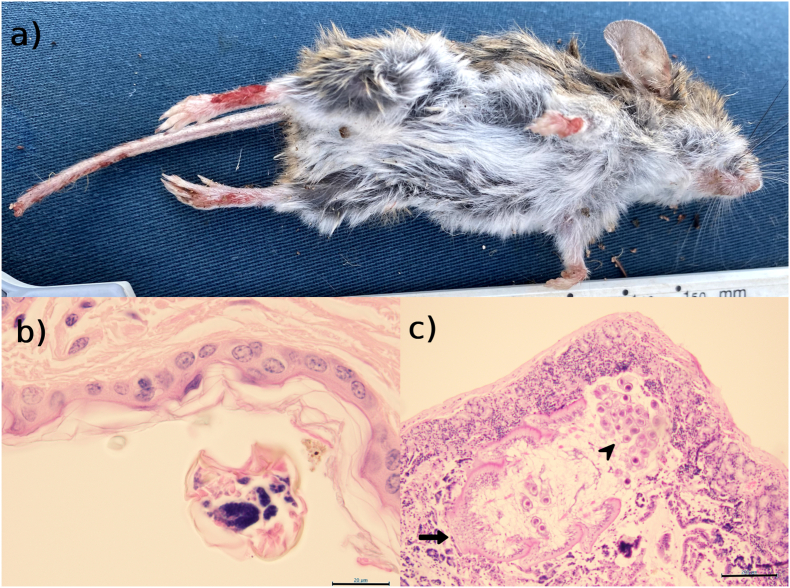
**Gross appearance and histopathological findings from the sandy inland mouse indicated multiple comorbidities. a)** Multiple bite wounds (presumed self-traumatic) are evident on the dorsal metatarsal and carpal regions, tail with traumatic amputation of the tip. **b)** a single metazoan is present overlying a focally hyperkeratotic epidermis (scale bar 20 μm). **c)***Hymenolepis microstoma* within a section of proximal duodenum, stained with H&E. The rostellum (arrow) is detached whilst eggs (arrowheads) are clearly visible (scale bar 200 μm).

Physical examination revealed small bite wounds present on the dorsal aspects of the metatarsals and carpi. The distal tail tip was missing, presumed traumatically amputated, and multiple bite marks were evident on the tail (Fig. 2a). Several ants (not identified) were found on the carcass, and multiple small full thickness skin tears were present on the back of the head as well as a <2 mm tear on the left lateral abdomen overlying the rib cage. No ectoparasites were visualised. The animal was in fair body condition (body condition score 2/5).

Significant findings on gross necropsy included the presence of an approximately 1 cm diameter mass in the central abdomen. The firm, spherical mass was tightly adhered to the colon and small intestine and had an irregular surface. Incision revealed a central core of caseous material. A heavy cestode burden was apparent in the duodenum with associated distension of the gastrointestinal tract, but no mucosal lesions were identifiable. The cervical salivary glands appeared subjectively enlarged along with associated lymph nodes. Similarly, the spleen, liver and kidney appeared subjectively enlarged.

### 3.2 Histopathology

On histopathological examination, the intra-abdominal mass appeared most consistent with a pancreatic exocrine adenocarcinoma. The mass was composed of a central focus of lytic necrosis surrounded by granulation tissue and in turn disorganised fibrous connective tissue in which numerous undifferentiated acini were present, which were occasionally seen to exhibit partial differentiation to exocrine pancreatic glandular epithelium. Rare mitoses were present (<1 per 10 high powered fields). A pronounced mixed inflammatory response (consisting of lymphocytes with fewer plasma cells, histiocytes, haemosiderophages and neutrophils admixed) was also present. Ziehl-Neelsen, PAS, and Gram histochemistry was negative for organisms.

The region of skin trimmed for histopathology was taken from a grossly normal site. A marginal, chronic, multifocal to coalescing lymphoplasmacytic and eosinophilic perivascular dermatitis was identified, with marginal multifocal orthokeratotic hyperkeratosis. A single transverse section of an arthropod was found overlying a focal area of hyperkeratosis (Fig. 2b). Additionally, an embryonated arthropod egg was embedded within a separate focal area of hyperkeratosis. Scybala (faeces) was present in some sections both on the epidermal surface and within the hyperkeratotic stratum corneum.

The lungs were unremarkable. A subjective increase in neutrophils was identified within the septal capillaries, but haematological confirmation of systemic neutrophilia and/or leukocytosis was not possible, as blood had not been collected. A mild multifocal subacute lymphocytic myocarditis was present, with multiple small lymphocytic aggregates visible. No organisms were visible and no significant cardiomyocyte degeneration or necrosis was present.

Other findings included a mild to moderate diffuse subacute lymphohistiocytic portal hepatitis, with mild, multifocal to coalescing, subacute biliary hyperplasia. The splenic red pulp was expanded by moderate extramedullary haematopoiesis. Aside from a single cestode visible within the proximal duodenum (Fig. 2c), the intestinal tract was unremarkable, although autolysis was noted. All other tissues (brain, kidney, testes, epididymis and salivary glands) were unremarkable.

### 3.3 Ancillary testing

**Fig. 3:**
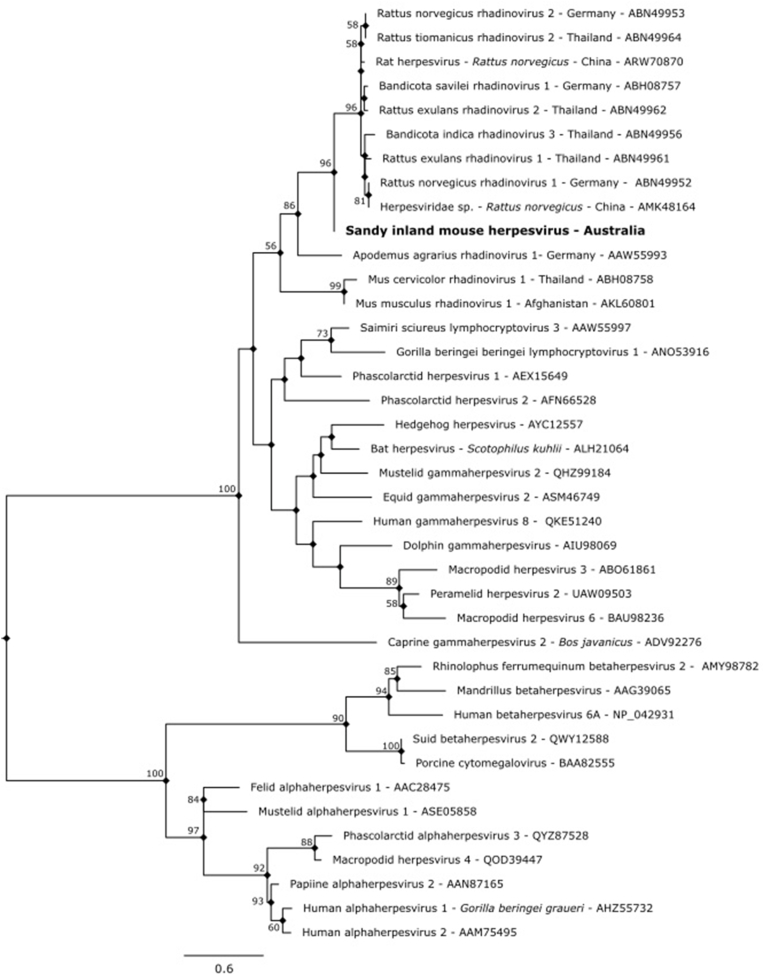
**Phylogenetic relationships of the putative novel herpesvirus identified in this case, on the basis of the amino acid sequence of the DNA polymerase catalytic subunit**. The evolutionary history was inferred by using the Maximum Likelihood method and Le and Gascuel model (Le and Gascuel, 2008). The percentage of trees in which the associated taxa clustered together is shown beside nodes (values > 50%). A discrete Gamma distribution was used to model evolutionary rate differences among sites (5 categories (+G, parameter = 3.2576)). The rate variation model allowed for some sites to be evolutionarily invariable ([+I], 4.35% sites). Branch lengths are measured in the number of substitutions per site. There were a total of 46 amino acid positions in the final dataset. Evolutionary analyses were conducted in MEGA11 (Tamura et al., 2021). The sequence length obtained was sufficient for characterisation as a member of the *Gammaherpesvirinae* subfamily, but further sequencing of additional gene regions would be necessary to determine generic assignment.

Sequencing of PCR products from both the conjunctival-oral and urogenital-rectal swabs identified a novel gammaherpesvirus sequence, tentatively designated as sandy inland mouse herpesvirus (SIMH). Both nucleotide and amino acid alignments consistently identified our sequence to be most closely aligned with rhadinoviruses previously identified in *Rattus* spp. internationally (Table 1, Fig. 3). However, in our case, the sequence length is insufficient for characterisation beyond the subfamily *Gammaherpesvirinae*.

**Table 1:** Results of basic alignment search (BLASTn) of nucleotide sequences obtained in this study, compared to the closest identified sequences obtained from GenBank. Nucleotide sequences have been uploaded to GenBank with accession numbers provided unless otherwise indicated.

Sample	Sequences obtained (GenBank accession number)	Closest described GenBank sequence (accession number)	Percent identity ^*(Query*^^*cover*^^*%)*^
**Oral-conjunctival and urogenital-rectal swabs**	Sandy inland mouse herpesvirus^a^	*Rattus norvegicus rhadinovirus* 2 (EF128039)	81.15^*(79%)*^
**Skin tissue**	*Sarcoptes scabiei* cox1 (Accession no PQ614282)	*Sarcoptes scabiei* (MF083739)	99.74^*(99%)*^
**Cestode**	*Hymenolepis* (syn. *Rodentolepis*) *microstoma* cox1 (Accession no PQ614279)	*Hymenolepis microstoma* (LR215992)	98.85^(*99%*)^
	*Hymenolepis* (syn. *Rodentolepis*) *microstoma* rrnS (Accession no PQ621271)		98.21^(*98%*)^

a The sequences obtained for the tentative sandy inland mouse herpesvirus can be found in Table S2.

The frozen skin section tested positive for *S. scabiei.* The generated nucleotide sequence of 398bp was closely aligned to other cox1 sequences of *S. scabiei* in GenBank, as identified through the BLASTn search. The closest alignment exhibited 99.74% identity across 99% of our nucleotide sequence (GenBank accession number PQ614282, Table 1). Phylogenetic analysis reinforced the clustering of our sequence with other *S. scabiei* cox1 gene sequences (Fig. S1). No attempt was made to test and sequence other gene regions.

Alignment of both the rrnS and cox1 gene regions amplified from the extracted cestode DNA, identified the cestode as *Hymenolepis* (syn. *Rodentolepis*) *microstoma* (Table 1, Fig. S2). Although no sequences of these gene regions were publicly available on GenBank for Australian-origin *H. microstoma*, sequences aligned closely with *H. microstoma* collected from diverse geographical regions (Fig. S2). Sequences have been uploaded to GenBank (GenBank accession numbers: rrns: PQ621271; cox1: PQ614279).

## 4 Discussion

This study reports a novel gammaherpesvirus, a new host-record for *H. microstoma,* and the first detection of *S. scabiei* in a sandy inland mouse. These findings provide important insights into our understanding of parasite presence for a species that has received limited research attention to date. They also contribute to important health baselines of rodent populations on DHI, prior to the commencement of a series of rodent translocations to the island, some of which have since taken place.

*Sarcoptes scabiei* is a zoonotic sarcoptiform mite of One Health importance, as the aetiological agent of both sarcoptic mange, which affects animal populations worldwide, and scabies, a neglected tropical disease of humans (El-Moamly, 2021; Escobar et al., 2022). Although *S*. *scabiei* has been reported from more than 148 species of mammal globally, including murid rodents (Khan et al., 2007; Escobar et al., 2022), to the authors’ knowledge, this is the first report in a wild Australian rodent. The pathogenesis of sarcoptic mange arises from both mechanical disruption and hypersensitivity responses induced by the epidermal burrowing life cycle of *S*. *scabiei* (Martin et al., 2018b; Næsborg-Nielsen et al., 2022)*.* Grossly our case demonstrated evidence of self-trauma, suggesting pruritis, and there was histopathological evidence of orthokeratosis and perivascular lymphoplasmacytic and eosinophilic dermatitis, albeit these changes were considered mild. However, associating *S. scabiei* to these abnormalities in our case is constrained by the histopathological examination of grossly normal skin and the lack of access to microscopy for immediate mite identification during necropsy.

Given the potential for *S. scabiei* to regulate and threaten wildlife populations, the detection of this parasite in a novel host in an island setting is significant (Martin et al., 2018a; Rudd et al., 2020; Ferreyra et al., 2022). However, our case was clearly moribund with multiple co-morbidities, which would increase susceptibility to infestation and disease expression of *S. scabiei*. Likely origins of *S. scabiei* in this case are speculative. Silent endemic infestation in the remnant small mammal community or human-mediated transmission (through previous wildlife translocations, fomites or temporary domestic animal landings on DHI) are plausible, but ultimately origins are unexplained (Arlian and Morgan, 2017; Browne et al., 2022; Escobar et al., 2022). Although whole genome sequencing may provide further insights into genetic relationships and thereby possible origins, it was not performed in this case due to a lack of regional sequences available from limited host species. No further cases of *S. scabiei* in wildlife on DHI have been detected since this report. However, ongoing monitoring for this parasite is essential, due to the dynamic nature of the disease and future introduction of potentially naïve hosts as part of wildlife translocations.

Another significant finding in our study is the detection of a partial novel *Gammaherpesvirinae* sequence, SIMH*.* Broader genome sequencing of this virus is needed to resolve its relationship with other members of the *Gammaherpesvirinae*. However, this is the first known report of a gammaherpesvirus (γHV) sequence detected from a native rodent in Australia, although this novelty likely reflects insufficient research attention provided to Australian native rodents. In the past two decades, several novel herpesviruses from both the *Beta*- and *Gammaherpesvirinae* subfamilies have detected in rodents worldwide, with both a high prevalence and diversity recorded across rodent species from Europe, Asia, the Americas, and Africa (Ehlers et al., 2007; Zheng et al., 2016; Ntumvi et al., 2018; Sotomayor-Bonilla et al., 2019). Australian native rodents may similarly host diverse herpesviruses, reflective of the host-specificity and co-evolution of herpesviruses with their natural hosts. The pathogenic implications and host range of the SIMH are currently unknown. Some gammaherpesviruses, including rodent γHVs may drive lymphoproliferative diseases, are oncogenic, and may reduce host fitness (Ackermann, 2006; Ambinder, 2000; Ehlers et al., 2007; Knowles et al., 2012; Wang et al., 2021), and whilst most exhibit a very narrow host tropism, broad host ranges and spillover to non-natural hosts has been documented for a limited number of γHVs, sometimes precipitating severe disease (Blasdell et al., 2003; Ackermann, 2006; Russell et al., 2009). Although presumably a co-evolved virus of sandy inland mice, further population level studies across different regions would be required to confirm both the natural host of SIMH and to determine any individual or population health impacts.

The identification of a substantial *H. microstoma* burden in the upper gastrointestinal tract of our case represents a new host-parasite association. *Hymenolepis microstoma* has previously been documented in house mice in Australia and has a global distribution, utilising several rodent species from different genera as definitive hosts, relying on various beetle intermediate hosts to complete its life cycle (Singleton et al., 1993; Cunningham and Olson, 2010). *Hymenolepis microstoma* may have indirect zoonotic potential, with molecular detection of *H. microstoma* eggs documented in human faeces in north-west Western Australia, although pseudoparasitism was not excluded (Macnish et al., 2003). Hepatic and biliary tract pathologies have been noted in experimental and natural infections of various rodent hosts, and the bile duct is the typical attachment site of *H. microstoma* documented in laboratory mice (Litchford, 1963; Hickman, 1964; Sanborn et al., 1970). In our case, given the duodenal location of the cestode (as also observed by Martins et al. (2022)), *H. microstoma* cannot be definitively said to have been the cause of the hepatobiliary lesions observed. Conversely, it also cannot be excluded as the cause, given physiologic discharge of the biliary tree into the duodenum may have resulted in its displacement to the site in which it was found. Given the broad host range of this parasite, it is likely that other omnivorous rodent species destined for translocation to Dirk Hartog Island, such as Shark Bay mice and desert mice, would be competent hosts for this tapeworm.

Overall, this case provides several critical insights into the baseline health of rodent populations on DHI, prior to rodent translocations that commenced in 2021. The detection of *S. scabiei* is particularly important, as the introduction of this pathogen into new locations and hosts may result in high morbidity and mortality, particularly in fragmented, isolated populations. Furthermore, the zoonotic potential of our findings emphasises the importance of human hygiene precautions during ecological monitoring programs. Further research is warranted to further explore the population health significance of the novel gammaherpesvirus detected in this study, and ongoing monitoring for *S. scabiei* in other wildlife on DHI is warranted. Ultimately, this case demonstrates the value of investigating mortalities and implementing health surveillance as part of ecological monitoring and wildlife translocation projects.

## CRediT authorship contribution statement

**Fiona Knox:** Writing – review & editing, Writing – original draft, Methodology, Investigation, Formal analysis, Conceptualization. **Nahiid Stephens:** Writing – review & editing, Methodology, Investigation. **Sarah Keatley:** Writing – review & editing, Methodology, Investigation. **Amanda Ash:** Writing – review & editing, Methodology, Investigation. **James Douch:** Writing – review & editing, Methodology, Investigation. **Saul Cowen:** Writing – review & editing, Supervision, Resources. **Kelly Rayner:** Writing – review & editing, Resources. **Rebecca Vaughan-Higgins:** Writing – review & editing, Supervision, Conceptualization.

## Funding

This work was supported by the 10.13039/100008190Holsworth Wildlife Research Endowment & The 10.13039/501100008702Ecological Society of Australia, the 10.13039/100026856Wildlife Disease Association – Australasia research award, and the Dirk Hartog Island Ecological Restoration Project funding.

The Dirk Hartog Island Ecological Restoration Project funding is provided through the Gorgon Barrow Island Net Conservation Benefits Fund.

In addition, Dr. Fiona Knox and Mr. James Douch were each supported by an Australian Government Research Training Program Scholarship, and the latter was also supported the National Taxonomy Research Grant Program.

## Declarations of competing interest

The authors declare no conflicts of interest.

## References

[bib1] Ackermann M. (2006). Pathogenesis of gammaherpesvirus infections. Vet. Microbiol..

[bib3] Algar D., Johnston M., Tiller C., Onus M., Fletcher J., Desmond G., Hamilton N., Speldewinde P. (2020). Feral cat eradication on Dirk Hartog Island, Western Australia. Biol. Invasions.

[bib4] Ambinder R.F. (2000). Gammaherpesviruses and “hit-and-run” oncogenesis. Am. J. Pathol..

[bib5] Andriantsoanirina V., Ariey F., Izri A., Bernigaud C., Fang F., Charrel R., Foulet F., Botterel F., Guillot J., Chosidow O., Durand R. (2015). *Sarcoptes scabiei* mites in humans are distributed into three genetically distinct clades. Clin. Microbiol. Infect..

[bib6] Arlian L.G., Morgan M.S. (2017). A review of *Sarcoptes scabiei*: past, present and future. Parasit. Vectors.

[bib7] Beckmann K.M., Cromie R.L., Sainsbury A.W., Hilton G.M., Ewen J.G., Soorae P.S., Kock R.A. (2022). Wildlife health outcomes and opportunities in conservation translocations. Ecolo. Solut. and Evid..

[bib8] Blasdell K., McCracken C., Morris A., Nash A.A., Begon M., Bennett M., Stewart J.P. (2003). The wood mouse is a natural host for *Murid herpesvirus 4*. J. Gen. Virol..

[bib9] Bowles J., Blair D., McManus D.P. (1992). Genetic variants within the genus *Echinococcus* identified by mitochondrial DNA sequencing. Mol. Biochem. Parasitol..

[bib10] Browne E., Driessen M.M., Cross P.C., Escobar L.E., Foley J., López-Olvera J.R., Niedringhaus K.D., Rossi L., Carver S. (2022). Sustaining transmission in different host species: the emblematic case of *Sarcoptes scabiei*. Bioscience.

[bib13] Cowen S., Sims C., Ottewell K., Knox F., Friend T., Mills H., Garretson S., Rayner K., Gibson L. (2023). Return to 1616: multispecies fauna reconstruction requires thinking outside the box. Animals.

[bib14] Cunningham L.J., Olson P.D. (2010). Description of *Hymenolepis microstoma* (Nottingham strain): a classical tapeworm model for research in the genomic era. Parasit. Vectors.

[bib15] Dickman C.R., Greenville A.C., Beh C.-L., Tamayo B., Wardle G.M. (2010). Social organization and movements of desert rodents during population "booms" and "busts" in central Australia. J. Mammal..

[bib16] Douch J.K., Devlin J.M., Whiteley P., Hartley C.A., Vaz P.K. (2022). Molecular detection of two new putative species of gammaherpesvirus in petaurid possums. Aust. Vet. J..

[bib17] Ehlers B., Küchler J., Yasmum N., Dural G., Voigt S., Schmidt-Chanasit J., Jäkel T., Matuschka F.R., Richter D., Essbauer S., Hughes D.J., Summers C., Bennett M., Stewart J.P., Ulrich R.G. (2007). Identification of novel rodent herpesviruses, including the first gammaherpesvirus of *Mus musculus*. J. Virol..

[bib18] El-Moamly A.A. (2021). Scabies as a part of the World Health Organization roadmap for neglected tropical diseases 2021-2030: what we know and what we need to do for global control. Trop. Med. Health.

[bib19] Escobar L.E., Carver S., Cross P.C., Rossi L., Almberg E.S., Yabsley M.J., Niedringhaus K.D., Van Wick P., Dominguez-Villegas E., Gakuya F., Xie Y., Angelone S., Gortázar C., Astorga F. (2022). Sarcoptic mange: an emerging panzootic in wildlife. Transbound. Emerg. Dis..

[bib20] Ferreyra H.D.V., Rudd J., Foley J., Vanstreels R.E., Martín A.M., Donadio E., Uhart M.M. (2022). Sarcoptic mange outbreak decimates South American wild camelid populations in San Guillermo National Park, Argentina. PLoS One.

[bib22] Hickman J. (1964). The biology of *Hymenolepis microstoma* (Dujardin). Pap. Proc..

[bib23] Khan A.M., Baruah H.C., Gogoi J.N., Mahanta J. (2007). Diagnosis | sarcoptic mange. Lab. Anim..

[bib24] Knowles S.C.L., Fenton A., Pedersen A.B. (2012). Epidemiology and fitness effects of wood mouse herpesvirus in a natural host population. J. Gen. Virol..

[bib25] Kock R.A., Woodford M.H., Rossiter P.B. (2010). Disease risks associated with the translocation of wildlife. Rev. Sci. Tech..

[bib26] Le S.Q., Gascuel O. (2008). An improved general amino acid replacement matrix. Mol. Biol. Evol..

[bib27] Litchford R.G. (1963). Observations on Hymenolepis microstoma in three laboratory hosts: *Mesocricetus auratus, Mus musculus*, and *Rattus norvegicus*. J. Parasitol..

[bib28] Macnish M.G., Ryan U.M., Behnke J.M., Thompson R.C. (2003). Detection of the rodent tapeworm *Rodentolepis* (=*Hymenolepis) microstoma* in humans. A new zoonosis?. Int. J. Parasitol..

[bib29] Martin A.M., Burridge C.P., Ingram J., Fraser T.A., Carver S. (2018). Invasive pathogen drives host population collapse: effects of a travelling wave of sarcoptic mange on bare-nosed wombats. J. Appl. Ecol..

[bib30] Martin A.M., Fraser T.A., Lesku J.A., Simpson K., Roberts G.L., Garvey J., Polkinghorne A., Burridge C.P., Carver S. (2018). The cascading pathogenic consequences of *Sarcoptes scabiei* infection that manifest in host disease. R. Soc. Open Sci..

[bib31] Martins N.B.G., del Rosario Robles M., Knoff M., Navone G.T., Callejón R. (2022). *Rodentolepis microstoma* isolated from different species of Sigmodontinae rodents (Rodentia: Cricetidae) in the Cuenca del Plata, Argentina: Morphological aspects and molecular characterization. Int. J. Parasitol. Parasites Wildl..

[bib32] Morris K., Page M., Kay R., Renwick J., Desmond A., Comer S., Burbidge A., Kuchling G., Sims C., Armstrong D.P., Hayward M.W., Moro D., Seddon P.J. (2015). Advances in Reintroduction Biology of Australian and New Zealand Fauna.

[bib33] Næsborg-Nielsen C., Wilkinson V., Mejia-Pacheco N., Carver S. (2022). Evidence underscoring immunological and clinical pathological changes associated with *Sarcoptes scabiei* infection: synthesis and meta-analysis. BMC Infect. Dis..

[bib34] Ntumvi N.F., Mbala Kingebeni P., Tamoufe U., Kumakamba C., Ndze V., Ngay Lukusa I., LeBreton M., Atibu Losoma J., Le Doux Diffo J., N'Kawa F., Takuo J.M., Mulembakani P., Nwobegahay J., Makuwa M., Muyembe Tamfum J.J., Gillis A., Harris S., Rimoin A.W., Hoff N.A., Fair J.M., Monagin C., Ayukekbong J., Rubin E.M., Wolfe N.D., Lange C.E. (2018). High herpesvirus diversity in wild rodent and shrew species in central Africa. Intervirology.

[bib35] Rudd J.L., Clifford D.L., Cypher B.L., Hull J.M., Jane Riner A., Foley J.E. (2020). Molecular epidemiology of a fatal sarcoptic mange epidemic in endangered San Joaquin kit foxes (*Vulpes macrotis mutica*). Parasit. Vectors.

[bib36] Russell G.C., Stewart J.P., Haig D.M. (2009). Malignant catarrhal fever: a review. Vet. J..

[bib37] Sanborn C.R., Marquardt W.C., Duszynski D.W. (1970). *Hymenolepis microstoma*: early histopathologic changes in mouse bile duct. Trans. Am. Microsc. Soc..

[bib38] Singleton G.R., Smith A.L., Shellam G., Fitzgerald N., Müller W.J. (1993). Prevalence of viral antibodies and helminths in field populations of house mice (*Mus domesticus*) in southeastern Australia. Epidemiol. Infect..

[bib39] Sotomayor-Bonilla J., Moreira-Soto A., Mendizabal D., Soley-Guardia M., Ramírez-Fernández J.D., Villalobos-Chaves D., Niehaus C., Gutiérrez-Espeleta G., Rico-Chávez O., Foley J., Suzán G., Chaves-Friedlander A. (2019). Diverse beta- and gammaherpesviruses in neotropical rodents from Costa Rica. J. Wildl. Dis..

[bib40] Tamura K., Stecher G., Kumar S. (2021). MEGA11: molecular evolutionary genetics analysis version 11. Mol. Biol. Evol..

[bib41] Trachsel D., Deplazes P., Mathis A. (2007). Identification of taeniid eggs in the faeces from carnivores based on multiplex PCR using targets in mitochondrial DNA. Parasitology.

[bib42] Ullman-Culleré M.H., Foltz C.J. (1999). Body condition scoring: a rapid and accurate method for assessing health status in mice. Lab. Anim. Sci..

[bib43] VanDevanter D.R., Warrener P., Bennett L., Schultz E.R., Coulter S., Garber R.L., Rose T.M. (1996). Detection and analysis of diverse herpesviral species by consensus primer PCR. J. Clin. Microbiol..

[bib44] Wang Y., Tibbetts S.A., Krug L.T. (2021). Conquering the host: determinants of pathogenesis learned from murine gammaherpesvirus 68. Annu. Rev. Virol..

[bib45] Wildlife Health Australia (2018).

[bib46] Yip S.J.S., Dickman C.R. (2023). Foraging and food selection in a desert rodent: diet shifts of the sandy inland mouse between population booms and busts. Animals.

[bib47] Zheng X.Y., Qiu M., Ke X.M., Zhou W., Guan W.J., Chen S.W., Li J.M., Huo S.T., Chen H.F., Jiang L.N., Zhong X.S., Xiong Y.Q., Ma S.J., Ge J., Chen Q. (2016). Molecular detection and phylogenetic characteristics of herpesviruses in rectal swab samples from rodents and shrews in southern China. Vector. Borne. Zoonotic Dis..

