# Supplementary material for: Making the most of mortalities: Novel host-parasite records in a sandy inland mouse (Pseudomys hermannsburgensis)
Source: Int J Parasitol Parasites Wildl. 2025 Jan 7;26:101037. doi: 10.1016/j.ijppaw.2025.101037 (PMC11786898; doi:10.1016/j.ijppaw.2025.101037)
Supplement: Multimedia component 1 [file mmc1.docx]

**Table S1:** Details of the primers and conditions utilised for PCRs reported in this study.

| **Taxon targeted** | **Gene region** | **Primer (5’–3’) and conditions** | **Reference** |
| --- | --- | --- | --- |
| ***Orthoherpesviridae*** | DNA polymerase catalytic subunit (UL30) | *Primary PCR*  DFA - GAYTTYGCNAGYYTNTAYCC  ILK - TCCTGGACAAGCAGCARNYSGCNMTNAA  KG1 - GTCTTGCTCACCAGNTCNACNCCYTT  *Nested PCR*  TGV - TGTAACTCGGTGTAYGGNTTYACNGGNGT  IYG - CACAGAGTCCGTRTCNCCRTADAT  *Conditions:*  Primary: 5 mins at 95°C, 45x: (30s at 95°C, 30s at 46°C, 90s at 72°C), 2 mins at 72°C  Nested: 5 mins at 95°C, 45x: (30s at 95°C, 30s at 46°C, 60s at 72°C), 2 mins at 72°C | VanDevanter et al. (1996) |
| **Cestoda** | 12S rrnS | Cest3    YGAYTCTTTTTAGGGGAAGGTGTG  Cest5    GCGGTGTGTACMTGAGCTAAAC  *Conditions:*  15 mins at 95°C, 40x: (30s at 94 °C, 90s at 58 °C, 10s at 72 °C), 7 mins at 72°C | Trachsel et al. (2007) |
|  | Cox1 | JB3 - TTTTTTGGGCATCCTGAGGTTTAT  JB4.5 - TAAAGAAAGAACATAATGAAAATG  *Conditions*  5 mins at 94°C 35x: (30s at 94 °C, 30s at 55 °C, 30s at 72 °C), 10 mins at 72°C | Bowles et al. (1992) |
| ***Sarcoptes scabiei*** | Cox1 | NavF - TGATTTTTTGGTCACCCAGAAG  NavR - TACAGCTCCTATAGATAAAAC  *Conditions*  5 mins at 94°C 35x: (30s at 94°C; 30s at 51°C, 40s at 72 °C), 7 mins at 72°C | Andriantsoanirina et al. (2015) |

**Table S2:** Nucleotide sequences obtained from amplification of the *Orthoherpesviridae* DNA Polymerase catalytic subunit (UL30) gene region in DNA extracted from both swabs collected from the sandy inland mouse.

| Oral-conjunctival swab | CGACGACCTTCGTCGGCCGGCGCATGCTGGGGGCTTCCATGGAAATGGTCAATTCCCTCTCGTTGGATCGACTCGAAGAGATCCACGGCCGTCCTCTGCCCCGGGAACCGGGGGCTTCGCTCCGCGTCATCTACGGNGACACGGACTCTGTGA |
| --- | --- |
| Urogenital-Rectal swab | CGCCNANNGACGACCTTCGTCGGCCGGCGCATGCTGGGGGCTTCCATGGAAATGGTCAATTCCCTCTCGTTGGATCGACTCGAAGAGATCCACGGCCGTCCTCTGCCCCGGGAACCGGGGGCTTCGCTCCGCGTCATCTACGGNGACACGGACTCTGTGA |

**
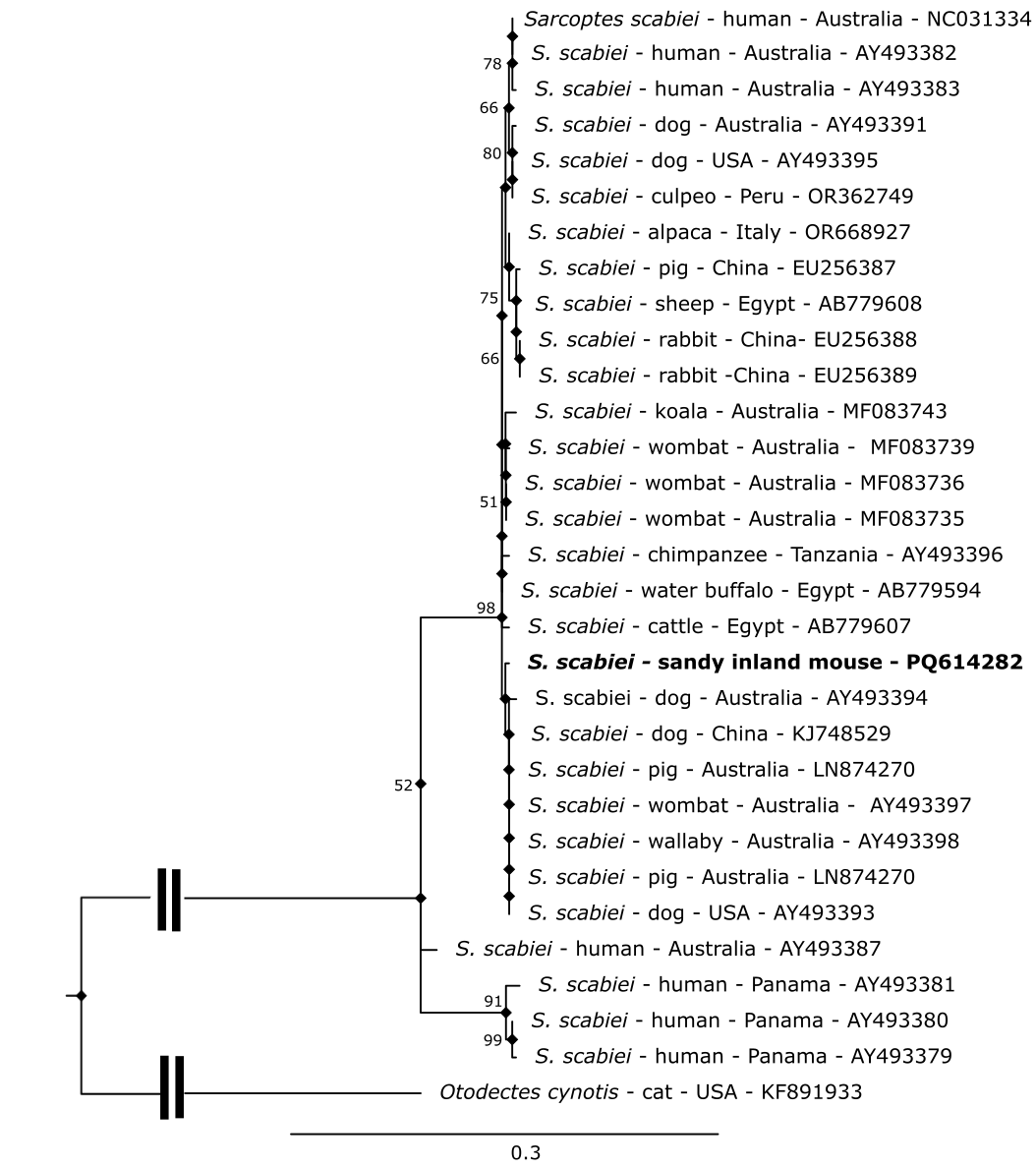
**

**Figure S1:** Phylogenetic tree indicating relationships of the *S. scabiei* cox1 nucleotide sequence identified in this case. The tree was constructed using the Maximum Likelihood method and Hasegawa-Kishino-Yano model (Hasegawa et al., 1985) with a discrete gamma distribution used to model evolutionary rate differences among sites. The sequence clustered closely with other *S. scabiei* cox1 sequences identified through GenBank. Bootstrap values >50% are indicated beside nodes. The scale bar represents the number of substitutions per site. There were a total of 387 positions in the final dataset.

**
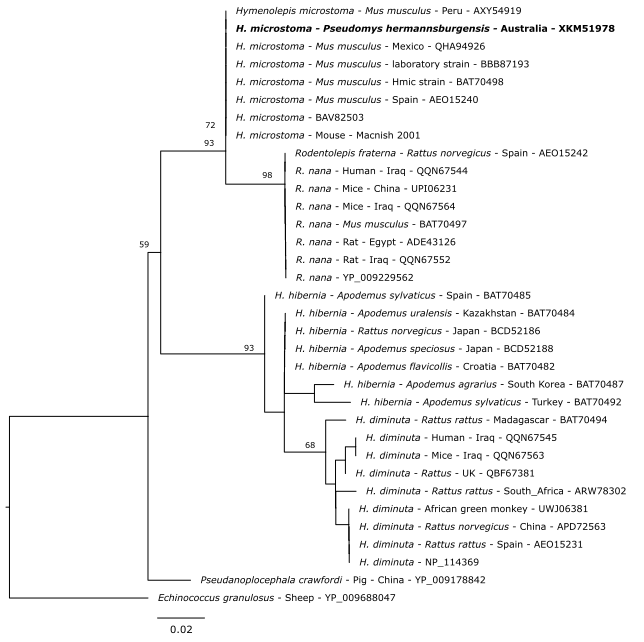
**

**Figure S2:** Phylogenetic tree indicating relationships of the cestode *Hymenolepis* (syn. *Rodentolepis*) *microstoma* cox1 amino acid sequence identified in this case, which clustered with other *H. microstoma* sequences obtained through GenBank. *Echinoccocus granulosus* (YP_009688047) was used as an outgroup. The tree was constructed using the Maximum Likelihood method and General Reversible Mitochondrial model (Adachi and Hasegawa, 1996) with a discrete gamma distribution used to model evolutionary rate differences among sites. Bootstrap values >50% are indicated beside nodes. There were a total of 120 amino acid positions in the final dataset. The scale bar represents the number of substitutions per site.
